# Role of adipose tissue macrophages in obesity-related disorders

**DOI:** 10.1084/jem.20211948

**Published:** 2022-05-11

**Authors:** Svetoslav Chakarov, Camille Blériot, Florent Ginhoux

**Affiliations:** 1 Shanghai Institute of Immunology, Shanghai JiaoTong University School of Medicine, Shanghai, China; 2 Institut Gustave Roussy, Batiment de Médecine Moléculaire, Villejuif, France; 3 Singapore Immunology Network, Agency for Science, Technology, and Research, Singapore, Singapore; 4 Translational Immunology Institute, SingHealth Duke-NUS Academic Medical Centre, Singapore, Singapore

## Abstract

The obesity epidemic has led researchers and clinicians to reconsider the etiology of this disease and precisely decipher its molecular mechanisms. The excessive accumulation of fat by cells, most notably adipocytes, which play a key role in this process, has many repercussions in tissue physiology. Herein, we focus on how macrophages, immune cells well known for their tissue gatekeeping functions, assume fundamental, yet ill-defined, roles in the genesis and development of obesity-related metabolic disorders. We first discuss the determinants of the biology of these cells before introducing the specifics of the adipose tissue environment, while highlighting its heterogeneity. Finally, we detail how obesity transforms both adipose tissue and local macrophage populations. Understanding macrophage diversity and their cross talk with the diverse cell types constituting the adipose tissue environment will allow us to frame the therapeutic potential of adipose tissue macrophages in obesity.

## Teaser

As the first immune cells to colonize tissues and long-lived resident cells, macrophages play important roles in tissue functions during early development, homeostasis, and disease. Here, Chakarov et al. discuss macrophage origin and functions in adipose tissue and how these features are modulated in obesity.

## Introduction

Obesity has become a global epidemic, with its occurrence nearly tripling since 1975. In 2016, >1.9 billion adults, accounting for 39% of the world’s adult population, were considered overweight, and >650 million were obese (World Health Organization, 2021). This has simultaneously led to an increase in obesity-linked diseases such as insulin resistance, type 2 diabetes (T2D), cardiovascular disease, nonalcoholic fatty liver disease, and cancer. Indeed, obesity and related disorders have long been considered metabolic diseases, but more recently, they have been associated with low-grade chronic inflammation and are starting to be regarded as inflammatory diseases driven by metabolic dysregulation (Dandona et al., 2004; Gonzalez et al., 2018; Johnson et al., 2012).

Considering such disorders as inflammatory in nature has many implications other than semantic issues, as it implies that their causes require reconsideration, and there is a need for a better comprehension of the underlying biological pathways. Inflammation is an immune response triggered by a wide variety of stimuli and involves the coordinated action of different immune cells (Medzhitov, 2008; Medzhitov, 2021). Among these cells, resident tissue macrophages (RTMs) have a central role as initiators of the process by sensing the initial immune assaults and, in response, producing a variety of inflammatory mediators such as cytokines and chemokines (Medzhitov, 2008). Macrophage biology is complex and modulated by several determinants that are briefly discussed below, including ontogeny, local environment/subtissular niches, and their niche-specific inflammatory status (Bleriot et al., 2020). Notably, macrophage origin can vary depending on conditions. It was assumed for decades that all RTMs derive from circulating monocytes (van Furth et al., 1972), but many studies have now revealed that the vast majority of RTM populations are actually established during embryonic development and subsequently self-maintained (Epelman et al., 2014; Ginhoux et al., 2010; Guilliams et al., 2013; Hoeffel et al., 2015; Schneider et al., 2014; Schulz et al., 2012). After birth, as tissues grow, circulating bone marrow (BM)–derived monocytes are recruited and contribute to the RTM pool in some tissues (Bleriot et al., 2020; Ginhoux and Guilliams, 2016; Liu et al., 2019; Scott et al., 2016). Therefore, every tissue contains a mix of RTMs that are either embryonically or BM derived. In addition to their ontogeny, the local environment—the so-called niche of residence—plays a part in influencing RTM diversity (Bleriot et al., 2020; Guilliams and Scott, 2017; Guilliams and Svedberg, 2021; Guilliams et al., 2020; Okabe and Medzhitov, 2014). However, the current niche concept often considers tissues as uniform units, and we have recently demonstrated that within each tissue type, subtissular niches exist that are populated by specific interstitial macrophage subpopulations—chemokine receptor CX3CR1^+^ and MHCII^+^ macrophages being in close contact with nerves and lymphatic vessel endothelial hyaluronan receptor 1 (LYVE1)^+^ macrophages associated with blood vessels containing smooth muscle cells (Chakarov et al., 2019; Lim et al., 2018).

Adipose tissue (AT), as the main body energy storage site, is impacted by obesity and has adapted to quickly respond to both caloric deprivation and excess (Rosen and Spiegelman, 2006). In healthy conditions, white adipose tissue (WAT) stores excess energy in the form of fat in a manner that is nontoxic to the cell (Rosen and Spiegelman, 2006). However, when energy intake consistently exceeds energy expenditure, the AT expands, mostly resulting from an increase in cell size (hypertrophy) but also from the recruitment of adipocyte precursors (hyperplasia; Ghaben and Scherer, 2019). These responses place demands on AT to dynamically react to the changing nutrient environment, a process generally referred to AT remodeling.

The regulation of energy uptake and fatty acid release by adipocytes are well understood at the molecular and cellular levels (Ghaben and Scherer, 2019). These actions require a supporting network of non-adipocyte cells, especially macrophages, but interactions within this supporting network, including those among macrophages and adipocytes, remain ill defined. Although the accumulation of macrophages in obese AT has been clearly described (Weisberg et al., 2003; Wellen and Hotamisligil, 2003; Xu et al., 2003), the precise functions in the development of the disease is less characterized, and a greater understanding is more necessary than ever. Herein, we discuss AT macrophage (ATM) heterogeneity and notably how this is modulated during obesity. We explore the cross talk that occurs between ATM and adipocyte/adipocyte precursors within subtissular niches and the changes in both cell subtypes during metabolic challenges.

## ATM heterogeneity in steady state and obesity

In addition to their so-called primary functions of tissue surveillance and dead-cell and debris clearance, RTMs perform several “accessory” functions in tissue homeostasis (Bleriot et al., 2020; Okabe and Medzhitov, 2016). These functions are believed to be specific to the tissue of residence and are driven by specific “master-regulator” transcriptional factors imprinted by tissue cues (Lavin et al., 2014). Notably, in the early 1980s, macrophages were hypothesized to play a role in energy metabolism in fat tissue (Kawakami et al., 1982; Pekala et al., 1983a; Pekala et al., 1983b). Here, we summarize the current understanding of ATM heterogeneity and their roles during obesity.

### In developmental and healthy conditions

#### ATM ontogeny

**Figure 1.**
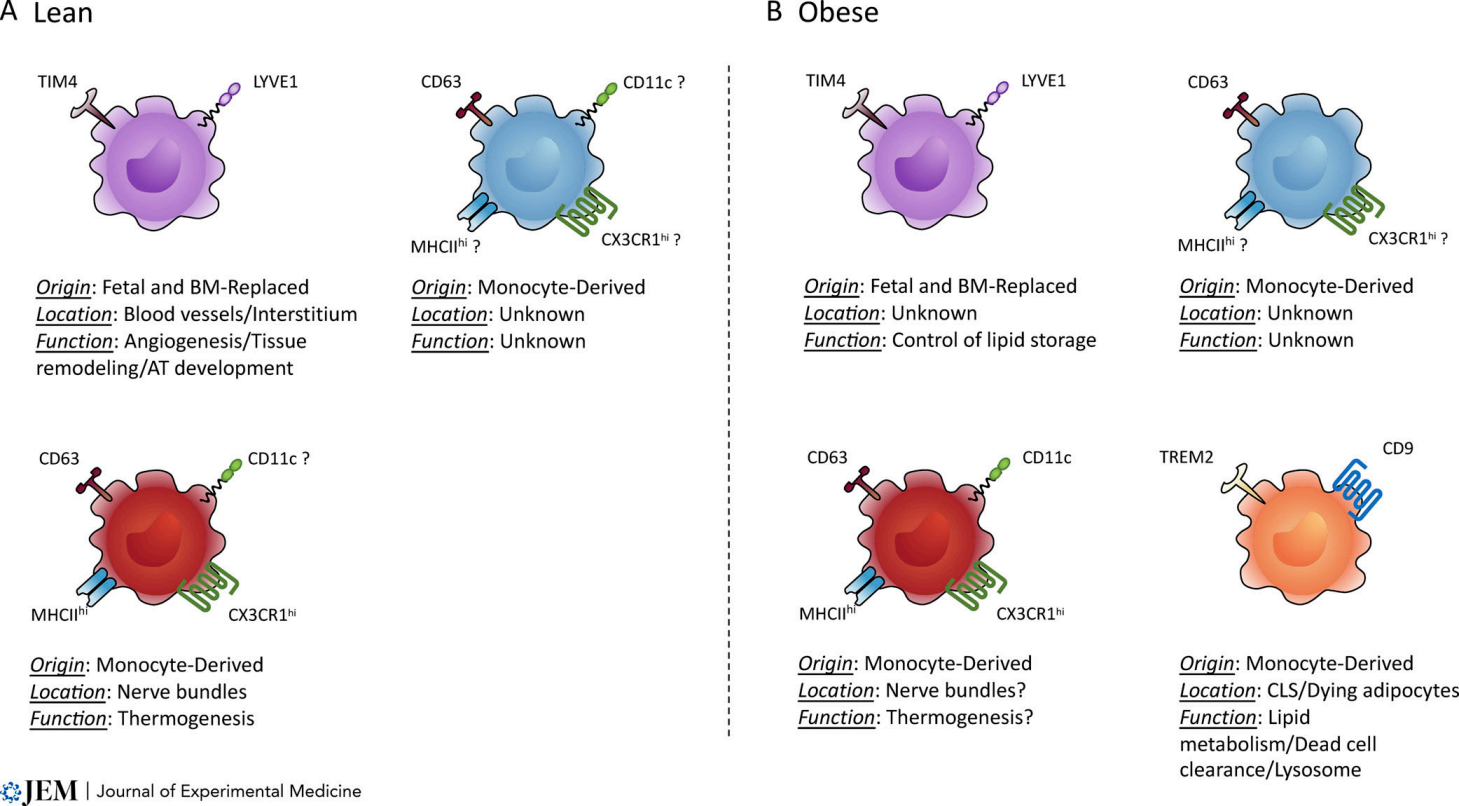
**AT macrophage subsets at steady state and obesity.** scRNA-seq, phenotypic, and functional studies identified specific subpopulations of ATMs at steady state and obesity. **(A)** In lean mice, three major populations of ATMs have been described. LYVE1^+^ ATMs are the first to populate the primitive AT. They are closely associated with vasculature and were reported to be of embryonic origin and slowly replaced by monocytes in adults. In addition, studies identified one or two CD63^+^ monocyte-derived ATM subpopulations based on differential expression of MHCII, CD11c, and CX3CR1. CX3CR1^hi^MHCII^hi^ were found predominantly associated with nerve bundles. However, the separation of the two monocyte-derived ATMs is still unclear, and CD11c expression can be attributed to both of them. **(B)** During obesity it was suggested that LYVE1^+^TIM4^+^ ATMs were implicated in control of lipid storage; however, their precise localization and origin during disease are still uncertain. In addition, the fate of the monocyte-derived ATMs from steady-state condition to obesity is not clear. With our current stage of knowledge, we placed LAMs, characterized with TREM2 and CD9 expression, as a separate population, as it is still under investigation if they are recruited during disease or acquire the LAM profile with obesity.

In lean steady-state AT, ATMs represent 5–10% of stromal cells (Weisberg et al., 2003) and appear as a heterogeneous population with diverse origins and functions (Chakarov et al., 2019; Cox et al., 2021; Hassnain Waqas et al., 2017; Jaitin et al., 2019; Silva et al., 2019; Fig. 1 A). The first evidence that ATMs can derive partially from embryonic progenitors came from observations of primitive epididymal WAT (eWAT) in neonatal mice. Those seminal studies reported the existence of a macrophage population expressing LYVE1 as early as 1 d after birth, when eWAT starts to form (Cho et al., 2007; Han et al., 2011; Wang et al., 2013). Interestingly, their presence in neonatal eWAT was stromal cell-derived factor 1 (SCF-1/CXCL12)–dependent but C-C chemokine receptor type 2 (CCR2)–independent, supporting their independence from circulating monocytes (Cho et al., 2007), which require CCR2 to egress from the BM (Kuziel et al., 1997; Serbina and Pamer, 2006). Later, using fate-mapping and parabiotic models, we and others confirmed the heterogeneity of ATMs by reporting two ontogenically distinct populations in steady-state murine tissues (Chakarov et al., 2019; Hill et al., 2018; Jaitin et al., 2019) based on LYVE1, MHCII, and CX3CR1 expression. These populations were found to derive from embryonic progenitors and are slowly replaced by monocyte-derived cells in adults (Chakarov et al., 2019). Of note, we also obtained evidence of a subtissular-specific localization for these subpopulations (Chakarov et al., 2019), with CX3CR1^+^MHCII^+^ ATMs being in close contact with nerves and LYVE1^+^ ATMs associated with blood vessels containing smooth muscle cells (Fig. 1 A).

Others studies have described four phenotypically different ATM populations, similar to the ones introduced above, including three vasculature-associated ones, based on T cell immunoglobulin- and mucin-domain-containing molecule (TIM4)–expressional variation in WAT (Silva et al., 2019) and MHCII and CX3CR1 expression in brown adipose tissue (BAT; Wolf et al., 2017). Given the relationship between TIM4 expression and the longer residency time of murine liver RTMs (Scott et al., 2016) or a specific population of embryonic-derived gut macrophages (De Schepper et al., 2018), it is likely that TIM4^+^ corresponds to long-lived embryonic ATMs. Indeed, recent studies have suggested there are three populations of ATM in subcutaneous WAT (scWAT) and eWAT based on the expression of TIM4, LYVE1, and CD11c (Cox et al., 2021; Magalhaes et al., 2021). Two TIM4^−^ populations with differential CD11c expression were decreased in Ccr2^−/−^ mice, suggesting their monocyte dependence. On the other hand, TIM4^+^ ATMs were labeled in both scWAT and eWAT using a yolk sac fate-mapping model based on the macrophage-specific expression of the colony-stimulating factor-1 receptor gene (*Csf-1r*; Cox et al., 2021) confirming their embryonic origin. In a similar approach using an inducible *Cx3cr1*-based fate-mapping model (Parkhurst et al., 2013), two independent studies concluded that murine perivascular ATMs were embryonically derived (Hassnain Waqas et al., 2017; Silva et al., 2019). The approach also revealed the tissue specificity of the cells by showing the different degrees of monocyte contribution toward WAT and BAT macrophages (Wolf et al., 2017). More recently, using a mouse model in which all monocyte-committed progenitors were labeled based on their specific expression of membrane-spanning 4-domains subfamily A member 3 (*Ms4a3*; Liu et al., 2019), we assessed the ontogeny of various ATM populations (Jaitin et al., 2019; Fig. 1 B). Notably, a population of triggering receptors expressed on myeloid cells 2 (*Trem2*)–expressing monocyte-derived ATMs recruited during obesity and harboring unique metabolic properties was identified (Jaitin et al., 2019).

#### Impact of ATM depletion

During eWAT development, LYVE1^+^ ATM recruitment provides a microenvironment with high levels of vascular endothelial growth factor α (VEGFα), matrix metalloproteinases (MMPs), and CXCL12, thus initiating angiogenesis and eWAT formation (Cho et al., 2007; Han et al., 2011). Depletion of VEGFα or whole-body macrophages using an injection of clodronate or CSF-1–blocking antibodies interferes with eWAT formation (Han et al., 2011) and adipocyte morphology (Wei et al., 2005). More interestingly, physical excision of the primitive eWAT and its inhabiting embryonic ATMs at postnatal day 4 impairs the development of mature AT in adults (Han et al., 2011), suggesting a fundamental role for these embryonic ATMs in eWAT formation, although the evidence is correlative. It was also shown that tribbles-homolog-1–deficient mice lacking several populations of macrophages, including BM, spleen, lung, and ATMs, develop metabolic syndrome and lipodystrophy (Satoh et al., 2013). Finally, the role of ATMs in AT development was also evidenced in *Csf-1r* mutant rats, supporting the aforementioned mouse-based discoveries (Cox et al., 2021; Pridans et al., 2018; Satoh et al., 2013). Taken together, these different knockout animal models have revealed that the presence of ATMs was required for AT development and homeostasis.

Despite the above findings, information on the precise mechanisms at play in such processes is still scarce. Using a *Tnfrsf11a*^Cre^*Csf-1r*^flox/flox^ mouse model, in which yolk sac macrophages are specifically depleted, the key roles of ATM-secreted platelet-derived growth factor cc (PDGFcc) were recently unraveled (Cox et al., 2021). The specific depletion of PDGFcc in macrophages during embryogenesis, or even systemically using blocking antibodies, inhibited eWAT development and lipid storage in adult mice (Cox et al., 2021). However, it is probable that other RTM populations, such as brain microglia, skin Langerhans cells, bone osteoclasts, or liver Kupffer cells (KCs), would also be affected in such a model. This highlights the importance of designing innovative tissue-specific model systems to avoid potential bystander and systemic effects. That aside, PDGFcc secretion by ATMs constitutes an experimental validation of the cell-circuit theory, which states that fibroblasts and macrophages form a stable two-cell circuit in which the density of each population is controlled by that of the other by way of the exchange of growth factors such as PDGF members (Zhou et al., 2018). In addition, in the healthy liver, liver RTMs, also named KCs, highly express *Pdgfc*, a gene that was gradually up-regulated when naive monocytes differentiated into mature KCs after depletion of embryonic KCs (Bonnardel et al., 2019). Recently, we added another layer to this complexity by showing that KCs do not actually form a homogeneous population: two subpopulations can be distinguished, one of which has a unique functional metabolic phenotype and expresses *Pdgfd*, another PDGF family member (Bleriot et al., 2021; De Simone et al., 2021). Thus, while we are still far from a global understanding, it is clear that macrophage-derived PDGF acts differentially in distinct niches depending on local signals.

### During obesity

**Figure 2.**
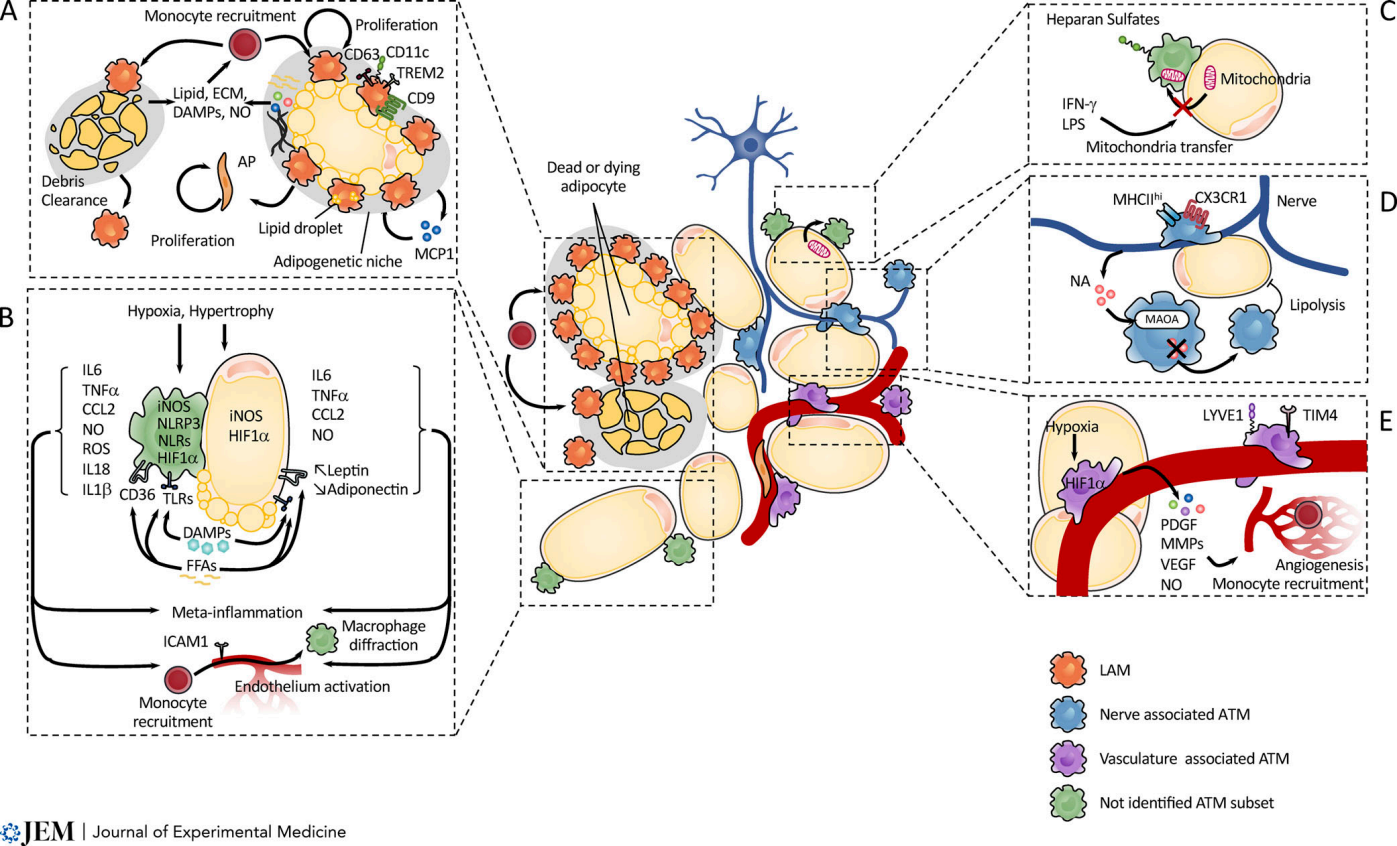
**eW-ATM subpopulations’ cross talk with their respective niches.** eWAT is populated by different subtypes of eW-ATM, with specific function and subtissue localization at steady state and obesity. **(A)** Crowning and LAMs are located surrounding dead or dying adipocytes, which release lipids and/or damage-associated molecular patterns (DAMPs), which in turn induce inflammation. Recruitment of LAMs also induces inflammation and ECM deposition and remodeling. The adipogenetic microenvironment created by cell death and macrophage recruitment in turn induces proliferation of both eW-ATM and AP, subsequently enhancing an inflammatory environment. **(B)** Hypoxia and adipocyte hypertrophy increase proinflammatory cytokine secretion in both macrophages and adipocytes through FFA release and DAMPs. These proinflammatory cytokines in turn increase obesity-associated meta-inflammation and monocyte recruitment/differentiation to macrophages. In addition to cytokines, adipocytes secrete adipokines such as Leptin and Adiponectin. Meta-inflammation dysregulation of Leptin and Adiponectin secretion is associated with increased recruitment of monocytes sustaining meta-inflammation. **(C)** A population of eW-ATMs involved in adipocyte-to-macrophage mitochondria transfer through heparan sulfate expression was identified. At steady state, this axis ensures maintenance of glucose and lipid homeostasis and is dysregulated during obesity in response to inflammation such as induces IFN-I and LPS. **(D)** Nerve bundles and sympathetic neuron-associated macrophages characterized with high CX3CR1 and MHCII expression are able to regulate lipolysis by capturing and degradation noradrenalin (NA) through monoamine oxidase A (MAOA). **(E)** LYVE1- and TIM4-expressing, embryonic derived eWAT-ATMs are found in contact with the eWAT vasculature. In response to hypoxia, they are able to produce angiogenic factors such as PDGF, MMP, and VEGF. **(E)** The supportive ECM-rich septum and fascia are endowed with distinct subpopulations of AP. However, to date it is still not known whether this specific subniche is endowed with specific eW-ATM subtypes controlling AP proliferation, differentiation, and recruitment.

Obesity induces a drastic increase in the proportion of ATMs, which reaches ≤40–50% of stromal cells in mouse (Weisberg et al., 2003) and human (Harman-Boehm et al., 2007) AT. To understand the role of this obesity-associated ATM accumulation, pioneering studies using osteopetrotic *Csf-1*^op/op^ mice carrying a homozygous missense mutation in the *Csf-1* gene (Weisberg et al., 2003; Wiktor-Jedrzejczak et al., 1990; Yoshida et al., 1990) and BM chimeras suggested that such obesity-associated ATMs were of monocytic origin (Weisberg et al., 2003). In addition, it was determined that *Ccr2*^−/−^ mice were protected from obesity-associated complications but not weight gain (Weisberg et al., 2006). Moreover, ATM number can also increase as a result of local proliferation (Kanda et al., 2006; Weisberg et al., 2006; Weisberg et al., 2003). The recruited monocyte-derived ATMs are often found forming crown-like structures (CLSs), in which they act as debris and lipid-droplet scavengers (Cinti et al., 2005; Sun et al., 2011). CLSs are suggested to preserve tissue integrity in the face of massive adipocyte cell death (McNelis and Olefsky, 2014) and are a potential niche for local ATM proliferation (Amano et al., 2014; Fig. 2 A).

#### Obesity-induced inflammation

ATM recruitment is linked to the chronic low-grade inflammation, also referred to as “meta-inflammation,” related to many of the comorbidities of obesity (Lumeng and Saltiel, 2011). Obesity-associated hypoxia was proposed as a key initiator of AT dysregulation (Trayhurn, 2013) and meta-inflammation, through up-regulation of proinflammatory mediators in adipocytes and macrophages, such as TNFα, IL-1, IL-6, CCL2, inducible nitric oxide synthase (iNOS), and others (Quintero et al., 2012; Wood et al., 2009; Ye et al., 2007). These various cytokines in turn amplify and sustain meta-inflammation by further recruiting, activating, and inducing macrophage proliferation and thereby driving glucose, lipid, and energy metabolic dysregulation (Hotamisligil, 2017; Reilly and Saltiel, 2017). The two main mechanisms implicated in monocyte recruitment to AT are pattern-recognition receptors on ATMs and chemokine secretion (Fig. 2 B). Free fatty acids (FFAs) are able to bind to both TLR4 and TLR2 (Lee et al., 2004; Nguyen et al., 2007), which in turn promote CCL2 secretion by adipocytes. TLR2 triggering by FFAs also induces cyclo-oxygenase-2 and iNOS expression by macrophages (Lee et al., 2004). Accordingly, TLR4^−/−^ and TLR2^−/−^ mice are protected from the inflammatory response and insulin resistance induced by obesity (Himes and Smith, 2010; Shi et al., 2006).

Dead and dying adipocytes are another contributing factor to meta-inflammation and monocyte recruitment (Fischer-Posovszky et al., 2011; Strissel et al., 2007). Damage-associated molecular patterns originating from damaged adipocytes are sensed by NOD-like receptors (NLRs; Jin and Flavell, 2013), which in turn activate NLRP3 inflammasomes in ATMs, inducing their secretion of IL-1β and IL-18 (Vandanmagsar et al., 2011). In addition, iNOS-driven NO secretion increases IL-1β production through NLRP3 inflammasome activation (Zhou et al., 2010; Fig. 2 B). As a consequence, NLRP3^−/−^ and iNOS^−/−^ animals are protected from obesity-related insulin resistance, macrophage infiltration, and extracellular matrix (ECM) deposition and are enriched in metabolically healthy adipocytes compared with WT mice (Becerril et al., 2018; Perreault and Marette, 2001; Stienstra et al., 2010). The high amount of NO produced by iNOS acts with reactive oxidative species, forming reactive nitrogen species and subsequently inducing nitrosative stress, which is a hallmark of inflammation (Foster et al., 2009; Furukawa et al., 2004; Kaneki et al., 2007). Not surprisingly, iNOS-induced nitrosative stress of the insulin-signaling pathway has emerged as a potent modulator of insulin resistance in obesity (Carvalho-Filho et al., 2005; Charbonneau and Marette, 2010; Perreault and Marette, 2001).

Although the studies cited above provided the framework for partially describing ATM-related functions during obesity, they have limitations stemming from their use of the outdated “M1/M2” macrophage classification proposed >20 yr ago (Mills et al., 2000). According to the model, M2 ATMs are associated with suppression of the immune response and ECM remodeling, are found in all AT depots, and are the dominant population in lean states (Lumeng et al., 2007). In contrast, a subtype of CD11c^+^ M1 ATMs was thought to be induced during obesity and to be associated with tissue damage and proinflammatory signaling (Lumeng et al., 2007; Lumeng et al., 2008). However, some studies have found evidence that remodeling capacity can be attributed more to the CD11c^+^ ATM subset than the resident ATM subset (Bourlier et al., 2008; Shaul et al., 2010). These contradictory observations are not surprising, given that the M1/M2 paradigm is an in vitro oversimplification, and we now know that these two polarizations represent only the extremes of a full spectrum of macrophage activation states (Ginhoux and Guilliams, 2016; Ginhoux et al., 2016). More recently, in addition to the M1/M2 ATM status, a “metabolic activated” ATM was proposed (Kratz et al., 2014), in which a metabolic layer can be added to the activation spectrum of ATMs.

#### ATM diversity during obesity

Single-cell RNA-sequencing (scRNA-seq) recently clarified the roles of different ATM subpopulations in obesity progression (Burl et al., 2018; Hildreth et al., 2021; Hill et al., 2018; Jaitin et al., 2019; Weinstock et al., 2019), describing ATM heterogeneity in a more unbiased manner. Thus, based on Ly6C and the tetraspanin CD9 expression, three ATM populations were described with variable CD11c expression (Hill et al., 2018). CD9^+^ ATMs, with a high intracellular lipid load, were found in CLSs and shown to induce inflammatory gene expression in AT of lean mice after adoptive transfer (Hill et al., 2018). Similarly, three ATM clusters were described (Jaitin et al., 2019), one of which, identified as CD9^−^CD63^−^ resident ATMs (or Mac1), comprised cells expressing genes associated with perivascular macrophages, such as *Retnla*, *Lyve1*, *Mrc1* (CD206), *Cd163*, and *Cd209f*, and were present in lean and obese animals (Chakarov et al., 2019; Jaitin et al., 2019; Lim et al., 2018). The other two CD9^+^ Mac2 and Mac3 clusters, which were more specific to the obese condition, were identified as infiltrating monocyte-derived ATMs using the *Ms4a3* model and were associated with CLSs (Jaitin et al., 2019; Figs. 1 and 2 A). Of note, similar populations were also found in human WAT (Hildreth et al., 2021). Further analysis of the differences between these three clusters identified Mac3 as the ATM implicated in lipid metabolism and phagocytosis and expressing genes such as *Trem2*, *Lipa*, *Lpl*, and *Cd36*, and thus named lipid-associated macrophages (LAMs; Jaitin et al., 2019). Importantly, LAMs were absent in *Trem2*^−/−^ mice, indicating that TREM2 may promote LAM program acquisition in ATMs (Figs. 1 and 2 A). Notably, in *Trem2*^−/−^ mice fed a high-fat diet (HFD), ATMs had a lower lipid content and failed to form CLSs, a phenotype correlated with adipocyte hypertrophy and increased weight gain. Interestingly, the LAM profile has been found by others in WAT (Burl et al., 2018; Hill et al., 2018), and in Alzheimer’s disease (Keren-Shaul et al., 2017), non-alcoholic steatohepatitis (Remmerie et al., 2020; Xiong et al., 2019), and fibrotic liver (Ramachandran et al., 2019), linking LAMs to a phenotype related to lipid uptake and storage, rather than specifically associated to obesity.

In addition, a population of murine ATMs involved in adipocyte-to-macrophage mitochondria transfer through heparan sulfate expression was recently reported (Brestoff et al., 2021). Interestingly, this axis was decreased during obesity, and myeloid cell-specific deletion of the heparan sulfate synthetic enzyme (*Ext1*) impaired mitochondrial uptake by macrophages, leading to dysregulated energy homeostasis and a susceptibility to diet-induced obesity in *Lyz2*^cre^-*Ext1*^flox/flox^ mice (Brestoff et al., 2021; Fig. 2 C).

In summary, beyond the M1/M2 paradigm, ATMs form a heterogeneous population of cells with distinct ontogeny, localizations, and function during tissue development, homeostasis, and disease. Ontogenically independent macrophage waves colonize fat tissue at different stages of development and in different states (including obesity) and end up localized to different subtissular niches that confer on them unique phenotype and functions (Bleriot et al., 2020; Guilliams and Scott, 2017; Guilliams and Svedberg, 2021; Guilliams et al., 2020). Thus, considering the niche as an important factor in ATM biology should provide a better understanding of AT anatomy and its changes during obesity.

## Dynamism of the AT niche during obesity

AT is a term encompassing heterogenous tissues with different functions that are distributed in specific anatomic locations across the body. Therefore, the different ATs constitute distinct environments and should potentially harbor different ATMs with unique identities and functions (Guilliams and Scott, 2017). Thus, in this section, we discuss AT anatomic differences and their specific ATM populations. We also discuss AT subtissular niches, proposing categorizations of eWAT subtissular niches and the changes occurring during obesity.

### Anatomically distinct niches

**Figure 3.**
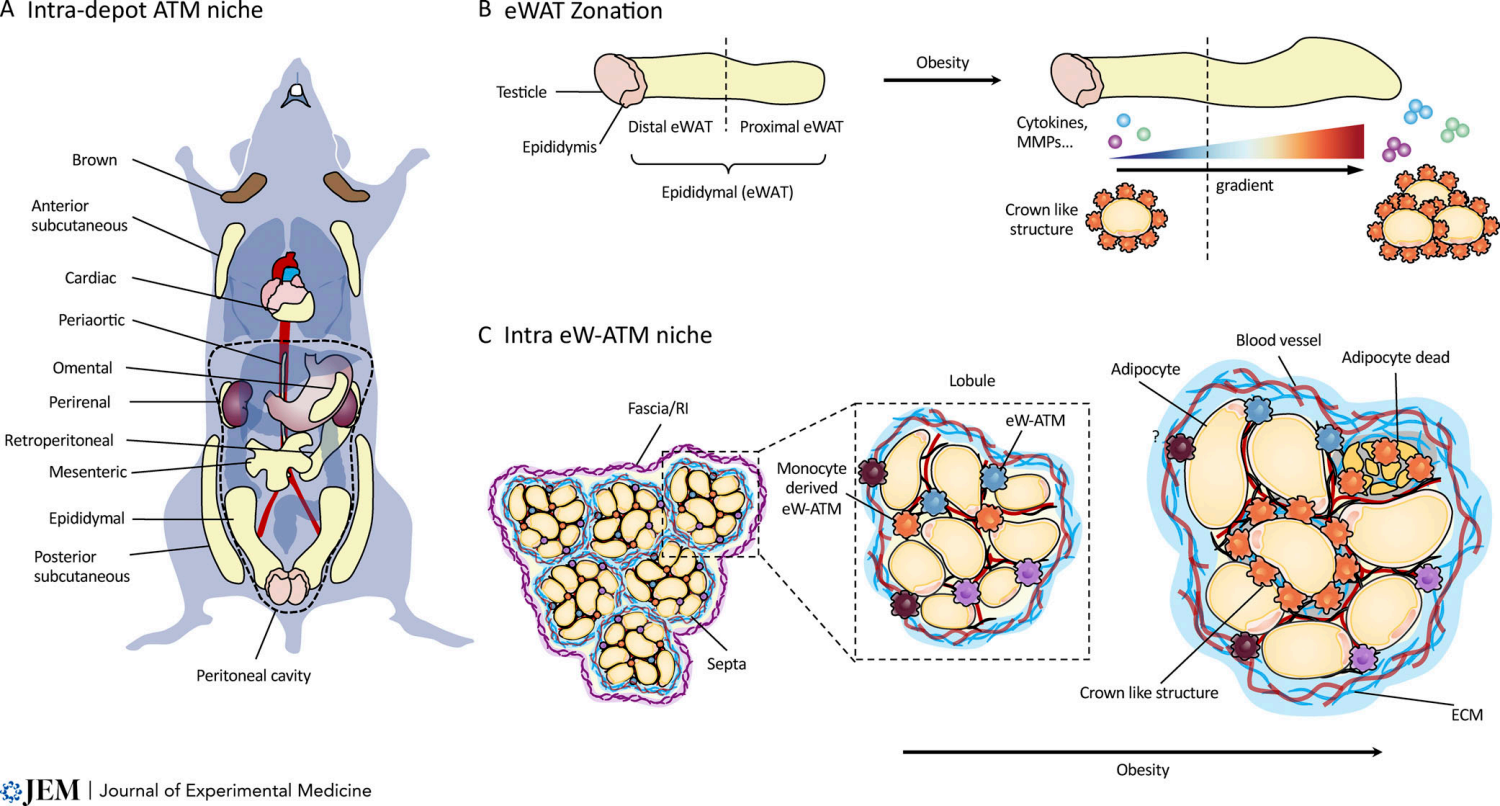
**Potential AT niches. (A)** Anatomic location of major ATs in mouse. The dotted line virtually indicates the peritoneal cavity. **(B)** eWAT was virtually subdivided in two zones, distal and proximal. During obesity, CLSs and adipokines were found enriched in the proximal zone. **(C)** eWAT is endowed with a well-organized stromal network and plastic architecture. eWAT plasticity is in part due to its organization into lobules, where mature adipocytes reside, and is delimited by bundles of ECM, or septa. Finally, the whole AT mass is surrounded by a capsule (also called the fascia or reticular interstitium [RI]) formed from an uninterrupted sheet of connective tissue that extends throughout the body.

In mammals, AT extends over the whole body and within distinct anatomic locations of fat deposition that confer distinct metabolic features on the tissue (reviewed in Chun [2021]; Fig. 3 A). Previous studies have typically considered WAT to be a homogeneous tissue, and scRNA-seq profiling has mostly been performed on visceral AT, i.e., eWAT (Hill et al., 2018; Jaitin et al., 2019; Weinstock et al., 2019) and scWAT (Burl et al., 2018). Therefore, the inherent complexity of the distinct AT depots has been widely overlooked in many immunologically focused studies so far.

#### AT ontogeny

From the developmental perspective, scWAT and BAT emerge perinatally, whereas most intra-abdominal depots appear after birth (Wang et al., 2013). In addition, scWAT and eWAT emerge from distinct mesenchymal lineages (Chau et al., 2014; Hepler and Gupta, 2017), whereas brown adipocytes and muscle cells share a common somatic origin, which is consistent with the finding that brown adipose cells express certain muscle-specific genes (Kajimura et al., 2015). Craniofacial, but not peripheral, scWAT depots originate from neuroectodermal rather than mesodermal structures (Billon and Dani, 2012). Moreover, the main difference between visceral WAT and scWAT is their draining system: scWAT and visceral WAT drain blood into the vena cava and hepatic portal vein, respectively (Shen et al., 2003). It has been suggested that this direct anatomic connection between visceral WAT and the liver explains the deleterious impact of visceral WAT in the promotion of liver steatosis and insulin resistance (Bjorntorp, 1990).

#### Major AT depot

Molecular and cellular studies have indicated that anatomically distinct depots are functionally and phenotypically different (Lee et al., 2013a; Macotela et al., 2012; Morgan-Bathke et al., 2015; Wu et al., 2012). The main difference between BAT and WAT is their function, i.e., the capacity to burn glucose and fat to produce heat in BAT versus energy storage in the form of intracellular triglycerides in WAT (Hepler and Gupta, 2017). It is interesting to consider also that the manner by which the AT expands is of great clinical significance during obesity (Hepler and Gupta, 2017). The preferential expansion of scWAT is associated with protection against cardiovascular diseases (Karpe and Pinnick, 2015; Lee et al., 2013a), whereas the expansion of visceral depots correlates with an increased risk of insulin resistance and T2D. Moreover, expansion through adipocyte hyperplasia is more favorable than that through hypertrophy (Lee et al., 2010), with hyperplasia being associated with adipose health and the delayed onset of insulin resistance (Gustafson et al., 2009). Interestingly, almost complete remodeling and adipocyte death can be observed in eWAT, but not scWAT, during HFD (Nishimura et al., 2008; Strissel et al., 2007). One explanation for this difference could be the preferential expansion of eWAT and scWAT by hyperplasia and hypertrophy, respectively (DiGirolamo et al., 1998). Accordingly, less macrophage infiltration and CLS formation is observed in scWAT compared with eWAT (Grove et al., 2010; Strissel et al., 2007), probably as a result of the local WAT depot–specific environments (Jeffery et al., 2016). Indeed, CCL2 was found to be released at higher levels in scWAT compared with eWAT in humans and mice (Bruun et al., 2005), suggesting there are different pathways of monocyte recruitment between these two AT depots. Finally, obesity-associated adipogenesis in male mice occurs specifically in eWAT, whereas in females, both gonadal WAT and scWAT undergo adipogenesis in a sex hormone–dependent manner (Jeffery et al., 2016). It is noteworthy to add that beyond its role in energy storage, eWAT, but not scWAT, is proposed to play a role in reproduction. Accordingly, excision of eWAT virtually eliminated spermatogenesis in male hamsters (Chu et al., 2010). Moreover, leptin—a hormone specifically secreted by AT—was associated with fertility (Donato et al., 2011). In addition to obesity and obesity complications, mice deficient in leptin (ob/ob; Zhang et al., 1994) or its receptor (db/db; Tartaglia et al., 1995) and humans deficient in the leptin receptor do not reach sexual maturity (Barash et al., 1996; Chehab et al., 1996; Farooqi et al., 2002). These examples add to the evidence of AT functional diversification.

### Distinct ATM in distinct AT depots

Considering the strong niche dependence of RTMs and the AT heterogeneity within an organism, a logical question arises: Do functionally and phenotypically distinct ATMs inhabit the different fat depots? Many hints to the answers have already been discovered. For example, as scWAT emerges during embryonic development, its ATM population unsurprisingly comprises more embryonic macrophages (Wang et al., 2013). In contrast, most eWAT appears after birth, and the monocyte contribution to ATMs is more pronounced (Wang et al., 2013). In obesity, the preferential expansion of scWAT is associated with protection against cardiovascular diseases, whereas the expansion of visceral depots correlates with an increased risk of insulin resistance and T2D, but the underlying roles of ATMs in these processes remain to be deciphered (Hepler and Gupta, 2017; Lee et al., 2013a).

At another level, the differential labeling of BAT and WAT macrophages in *Cx3cr1*^Cre-ERT2^-*Rosa*^tdTom^ mice suggested that BAT has a higher proportion of CX3CR1^+^ nerve-associated ATMs (Chakarov et al., 2019) and slower monocyte turnover (Wolf et al., 2017). In addition, targeted deletion of the nuclear transcription regulator methyl-CpG-binding protein 2 gene in CX3CR1^+^ BAT ATMs induced spontaneous obesity linked to an altered homeostatic energy expenditure and impaired thermogenesis (Wolf et al., 2017). Similarly, the CX3CR1^+^ sympathetic neuron-associated macrophage population controls WAT browning through norepinephrine clearing, subsequently controlling weight gain and thermogenesis (Pirzgalska et al., 2017; Fig. 2 D). In WAT, obesity-associated hypertrophy and adipocyte progenitor (AP) proliferation were shown to be under the control of LAMs and the total ATM population, respectively (Jaitin et al., 2019; Nawaz et al., 2017). Inhibiting or blocking PDGFcc expression resulted in embryonic macrophages failing to support lipid storage and synthesis during obesity-associated weight gain (Cox et al., 2021), while in BAT, monocyte recruitment controls tissue expansion through podoplanin engagement (Gallerand et al., 2021). In wound beds, dWAT AP proliferation is specifically supported by distinct CD206^+^CD301b^+^ wound-associated macrophages during healing (Shook et al., 2018).

In conclusion, the simple designation of WAT as “subcutaneous” or “visceral” tissue might be too simplistic, and as a consequence, the designation of macrophages as “adipose tissue macrophages” is then also an oversimplification. Different macrophage populations and depot-specific macrophage subpopulations with distinct functions and metabolic properties exist and need to be further defined and explored. Here, we propose a change to the terminology, with the designation of specific names for the anatomically distinct ATMs. In this model, eWAT macrophages are called eW-ATMs, scWAT macrophages are scW-ATMs, BAT macrophages are B-ATMs, and so on, as this terminology will facilitate the identification and functional characterization of ATMs.

### Intra-AT niche and its remodeling during obesity? The example of eWAT

Abdominal WAT is composed of six different fat depots surrounding specific anatomically distinct regions of the abdomen (Chun, 2021; Fig. 3 A). Because of its abundance, eWAT is the most broadly studied intra-abdominal WAT. In its position attached to the testicles and epididymis in males and gonads in females, eWAT then extends toward the diaphragm (Chun, 2021), and it is virtually subdivided into two—distal and proximal—(Tirard et al., 2007) or three zones—medial, caudal, and rostral—using spermatic blood vessels as a hallmark (Altintas et al., 2011; Fig. 3, A and B).

#### eWAT zonation

In vertebrates, AT is the only tissue able to change its mass by a substantial amount even after it reaches its adult size, which plays a crucial role in controlling energy storage and release (Hausman et al., 2001; Spiegelman and Flier, 1996). Interestingly, during long-term HFD-induced obesity, CLS formation is increased in the apical or rostral part of the eWAT (Altintas et al., 2011), indicating the occurrence of eWAT zone–dependent monocyte recruitment during HFD (Fig. 3 B). Similar eWAT zonation has been proposed in other reports, where it is subdivided into the distal zone (corresponding to the rostral zone described in Altintas et al. [2011]) and the proximal zone (which is in close contact with the testicles and epididymis; Lee and Kim, 2019; Tirard et al., 2007). There were differences found in aldo-keto reductase 1B7 detoxification enzyme between the distal and proximal eWAT (Tirard et al., 2007) and specific age-associated increased expression of adipocyte-related genes such as *Fabp4*, *Lpl*, *Dlk1*, *Pparg*, *Lep*, and *Adipoq* in distal eWAT (Lee and Kim, 2019; Tirard et al., 2007), suggesting that possible zone-associated eW-ATM niches may exist which need to be further characterized (Fig. 3 B).

At the microscopic level, eWAT is endowed with well-organized stromal, nerve, and immune networks. The main tissue mass is composed of white adipocytes; however, they represent only ∼20% of the total cellularity of eWAT (Eto et al., 2009; Rondini and Granneman, 2020). The remaining 80% of the tissue includes well-defined fibroblasts, vascular cells, immune cells, and APs (Eto et al., 2009; Prunet-Marcassus et al., 2006; Rondini and Granneman, 2020), each of which contributes to the synthesis of and changes to the ECM, which is implicated in eWAT remodeling in response to nutrient excess or deficiency. This is in part due to the eWAT plastic architecture, which is organized into lobules, or stroma, where mature adipocytes reside (Esteve et al., 2019) and is delimited by bundles of ECM, or septa, composed of collagen, fibronectin, elastin, and hyaluronan (also known as hyaluronic acid or HA;Esteve et al., 2019; Grandl et al., 2016; Kang et al., 2013; Spiegelman and Ginty, 1983). Finally, the whole AT mass is surrounded by a capsule (also called the fascia or reticular interstitium) formed from an uninterrupted sheet of connective tissue that extends throughout the body (Benias et al., 2018; Su et al., 2016; Fig. 3 C).

#### Vascular niche

All AT depots including eWAT are highly vascularized (Hausman and Richardson, 2004). During eWAT development, angiogenesis precedes adipogenesis (Han et al., 2011; Hausman and Richardson, 2004), and LYVE1^+^ vasculature-associated macrophages are recruited to primitive eWAT, even before adipocyte development, supporting the formation of a dense vascular network that is induced by VEGFα and MMP expression (Cho et al., 2007; Han et al., 2011; Lim et al., 2018). During adulthood, a population of blood vessel–associated macrophages with high endocytic functions is present (Fig. 2 D; Chakarov et al., 2019; Silva et al., 2019). It was recently proposed that the recruitment of eW-ATMs can control vascular integrity during HFD (Chen et al., 2021). Indeed, obesity is characterized by rapid adipocyte expansion and accumulation, thereby drastically affecting AT vascularization, leading to hypoxia and poor vascular system maintenance and growth (Hodson et al., 2013). Subsequently, a drastic drop in AT oxygenation was observed in rodents and patients (Gonzalez-Muniesa et al., 2015), leading to the activation of hypoxia-inducible factor (HIF) signaling (Wood et al., 2009). In turn, HIF1α increases the production of angiogenic factors such as VEGFα and angiopoietin-2 (Tahergorabi and Khazaei, 2013) and induces inflammation (Murdoch et al., 2005), drastically affecting the ATM vascular niche (Fig. 2 E). As a consequence, deletion of HIF1α in adipocytes prevents obesity-associated inflammation and complications (Lee et al., 2014). Regarding macrophages, HIF signaling in eW-ATMs increases their PDGF secretion, which in turn stimulates capillary formation (Pang et al., 2008), probably by stimulating the related receptor expressed by endothelial cells (Greenberg et al., 2008). HIF1α also stimulates NO production by inducing iNOS expression in macrophages (Takeda et al., 2010), thereby promoting angiogenesis (Fig. 2 D).

#### ECM niche

eWAT blood vessel development/maturation and ECM deposition are tightly linked processes. Dense connective tissue contains immature capillary beds and few adipocytes, whereas loose connective tissue contains mature capillary beds surrounded by more adipocytes (Crandall et al., 1997). In addition, the density of the ECM gradually increases with age from birth to adulthood (Hausman and Kauffman, 1986). Accordingly, in obesity, the angiogenic capacities of AT decrease in a manner linked to obesity-associated metabolic dysfunction (Spencer et al., 2011), which correlates with dense ECM depositions of material such as HA and collagen VI (Kang et al., 2013; Khan et al., 2009) and fibrosis (Sun et al., 2011). Therefore, putative ECM and blood vessel eW-ATM subtissular niches are highly plastic, and during obesity, eW-ATMs are implicated in ECM remodeling itself. Thus, macrophage-enriched CLSs surrounding dying adipocytes are associated with ECM deposition (Cinti et al., 2005). It has been suggested that elastin-associated LYVE1^+^ eW-ATMs are the main cells producing MMP-12 and MMP-9 and, thus, play an active role in elastin remodeling (Lim et al., 2018; Martinez-Santibanez et al., 2015). Moreover, macrophage-derived proinflammatory signals may activate ECM-related genes in APs (Keophiphath et al., 2009). In addition, low-grade inflammation induces the upregulation of macrophage-inducible C-type lectin (Mincle) and is implicated in macrophage-dependent ECM deposition. Accordingly, *Mincle*^−/−^ mice are protected from obesity-associated CLS formation. Moreover, eW-ATMs participate in collagen uptake involving CD206, urokinase plasminogen activator receptor-associated proteins Endo180 and Mrc2, or MFGE8 (Atabai et al., 2009; Madsen et al., 2013; Tanaka et al., 2014).

#### Mature adipocytes and CLSs

Approximately 10% of adipocytes are renewed annually by continuous turnover from APs, and decreased regeneration rates are associated with AT hypertrophy (Arner et al., 2010; Spalding et al., 2008). To maintain homeostasis, old apoptotic adipocytes are removed by macrophages (Duvall et al., 1985; Keuper et al., 2011; Suganami et al., 2007). During obesity, necrosis of adipocytes driven by hypertrophy is a prominent phagocytic stimulus that regulates ATM infiltration, which is gradually increased (Cinti et al., 2005), and macrophage–adipocyte interaction was proposed to aggravate meta-inflammation (Wellen and Hotamisligil, 2003; Fig. 2, A and B).

CLSs are hallmarks of obesity, and ≤90% of macrophages are localized within CLSs in obese Leptin-deficient (db/db) mice and obese patients (Cinti et al., 2005). The microenvironment created by CLSs contributes to macrophage proliferation during HFD feeding (Haase et al., 2014), and in later stages of obesity, AT expansion was associated with increased CLS formation and adipocyte death (Coats et al., 2017; Geng et al., 2021). Meanwhile, eW-ATM accumulation correlates with CLS formation, implicating CLSs as a possible contributing event to eW-ATM accumulation (Weisberg et al., 2003). CLSs metabolically reprogram eW-ATMs through their lipid-rich microenvironment, thereby increasing eW-ATM lipid content (Fig. 2, A and B). This process is mediated via upregulation of surface molecules such as ABCA1, very low-density lipoprotein receptor, perilipin, macrophage receptor with collagenous structure, and class B scavenger receptor CD36 (Brunner et al., 2020; Huang et al., 2014; Kratz et al., 2014). Moreover, FFAs increase the uptake of lipids through lysosomal exocytosis detected by increased LAMP1 and LAMP2 in eW-ATMs from obese mice (Coats et al., 2017).

Basal levels of FFAs increase during obesity, and in addition to TLRs, as discussed above, FFAs bind CD36 receptor, also expressed by macrophages (Fig. 2 B). FFA-bound CD36 triggers eW-ATM activation, and accordingly, CD36^−/−^ mice display improved insulin sensitivity and reduced AT inflammation upon HFD (Kennedy et al., 2011; Kuda et al., 2011; Fig. 2 B). Moreover, macrophage-targeted silencing of lipid handling genes—lipoprotein lipase and CD36—decreases lipid load in eW-ATMs, correlating with an increase of circulating FFAs (Aouadi et al., 2014). On the contrary, enhancing the lipid storage capability of ATMs by depletion of lysosomes with chloroquine treatment decreases basal lipolysis and serum FFAs in obese mice (Xu et al., 2013; Fig. 2 A). Accordingly, a lipid-buffering subset of eW-ATMs was described by Jaitin et al. (2019), interacting with adipocytes within CLS and facilitating lipid droplet phagocytosis throughout TREM2 engagement. CLS-associated, lipid-laden eW-ATMs are also characterized by the high expression of CD11c and are defined as monocyte derived. It is suggested that CLS-associated eW-ATMs facilitate adipocyte progenitor differentiation and/or proliferation through osteopontin secretion (Lee et al., 2013b; Nawaz et al., 2017; Fig. 2 A).

Adipocytes also link the innate immune system through secretion of cytokines, lipid, and hormones, mediators regrouped under the name of “adipokines” (Blaszczak et al., 2021; Galic et al., 2010; Kershaw and Flier, 2004; Tilg and Moschen, 2006). Since the definition of AT as an endocrine organ, >50 adipokines secreted by adipocytes have been described (Recinella et al., 2020; Rodriguez et al., 2015) that are able to modulate physiological functions such as body weight, appetite, glucose homeostasis, and blood pressure, as well as inflammation and the immune system (Blaszczak et al., 2021; Mancuso, 2016; Trujillo and Scherer, 2006; Wang and Scherer, 2016). Some of them, such as Leptin, Adiponectin, serum amyloid A (SAA), HA, FFAs, and many others, play roles in eW-ATM accumulation and meta-inflammation (Bai and Sun, 2015; Fig. 2 B).

In particular, obesity is associated with increased levels of Leptin (Tilg and Moschen, 2006) involved in both innate and adaptative immunity and macrophage recruitment (La Cava and Matarese, 2004). In addition, mice deficient in Leptin (ob/ob) or Leptin receptor (db/db) show a decrease of macrophage recruitment in AT despite increased weight gain and adiposity (Dib et al., 2014; Xu et al., 2003). Leptin also upregulates proinflammatory cytokine expression such as TNFα and IL6 (Lopez-Jaramillo et al., 2014), as well as adhesion molecules on endothelial cells (Curat et al., 2004), both implicated in inflammation and macrophage recruitment (Fig. 2 B).

Unlike Leptin, Adiponectin, an adipokine involved in energy homeostasis, is reduced in obesity (Kern et al., 2003) and possesses anti-inflammatory properties on macrophages (Ohashi et al., 2010; Ouchi et al., 2001; Qi et al., 2014). Inversely, macrophage-derived TNFα during obesity inhibits Adiponectin levels in AT, whereas in lean state, Adiponectin inhibits macrophage “foam cell” formation as well as endothelial cell activation and monocyte recruitment (Sikaris, 2004; Fig. 2 B).

Other adipocyte-derived factors, such as SAA and HA, facilitate monocyte adhesion through increase of chemotaxis. Thus, SAA is secreted by adipocytes and macrophages in response to inflammation (Lindhorst et al., 1997), stimulating intercellular adhesion molecule 1 expression on endothelial cells and thereby promoting monocyte recruitment (Mullan et al., 2006; Fig. 2 B).

## Concluding remarks

While we extensively discussed macrophage biology, it is obviously necessary to obtain in the future more detailed profiles of the other cell types present in ATs, including stromal, vascular, and other immune cells, such as innate lymphoid cells (ILCs), T cells, eosinophils, monocytes, or neutrophils. ILC2 cells, for example, promote the proliferation of eosinophils through IL5 secretion. In turn, the IL4 produced by eosinophils, together with IL13 from ILC2, imprints an eW-ATM phenotype and promotes peroxisome proliferator-activated receptor γ expression, as well as the proliferation of PDGFRα^+^ stromal cells. This illustrates the importance of the local microenvironment in maintaining AT homeostasis and the absolute need to consider all elements of the microenvironment when attempting to understand disease development.

Deciphering obesogenic mechanisms will require a similarly deep understanding of AT biology but also an exploration of the cross talk that occurs between ATs and other organs harboring metabolic functions. It would be simplistic to consider obesity only as a hypertrophy and/or hyperplasia of ATs. Related disorders are observed in several organs, such as the pancreas (which regulates insulin secretion; de Oliveira et al., 2020), muscles (Pedersen and Febbraio, 2012), and the liver (Diehl, 2010), and obesity appears, therefore, to be a global condition rather than a tissue-specific disease (Gesmundo et al., 2021). Obesity often results in non-alcoholic fatty liver disease, the more severe non-alcoholic steatohepatitis, or even fibrosis or cirrhosis (Diehl, 2010). These inflammatory diseases are implicated in hepatocarcinogenesis (Sun and Karin, 2012), and a strong link between obesity and liver cancer has been identified (Larsson and Wolk, 2007). Hence, it appears that extensively studying all metabolic organs is fundamental to understanding obesity.

Another interesting dimension to consider further is the effect of maternal imprinting on the AT niche in offspring. Indeed, according to recent National Health and Nutrition Examination Survey data (Skinner et al., 2018), a sharp increase in the prevalence of severe obesity among 2–5-yr-old children has been observed, and parental dietary habits have been clearly linked to this. Recent studies demonstrated that maternal HFD-induced long-term sex-dependent obesity in offspring relates to the metabolic reprogramming of adipocyte differentiation and hypertrophy (Chang et al., 2019; Liang et al., 2016; Litzenburger et al., 2020). Conversely, maternal exercise favors the browning of WAT and BAT during formation, preventing metabolic disfunction in offspring upon HFD challenge (Son et al., 2020). Because of the importance of ATMs in diet-induced obesity and the alarming rise in global cases of obesity, maternal HFD imprinting in offspring ATMs urgently needs to be investigated.
